# Implications for registry-based vaccine effectiveness studies from an evaluation of an immunization registry: A cross-sectional study

**DOI:** 10.1186/1471-2458-8-160

**Published:** 2008-05-14

**Authors:** Barbara E Mahon, Kimberly M Shea, Nancy N Dougherty, Anita M Loughlin

**Affiliations:** 1 Department of Epidemiology, Boston University School of Public Health, Boston, MA, USA; 2 Department of Pediatrics, Boston University School of Medicine, Boston, MA, USA; 3 Department of Epidemiology, Johns Hopkins Bloomberg School of Public Health, Baltimore, MD, USA; 4 Novartis Vaccines, Cambridge, MA, USA

## Abstract

**Background:**

Population-based electronic immunization registries create the possibility of using registry data to conduct vaccine effectiveness studies which could have methodological advantages over traditional observational studies. For study validity, the base population would have to be clearly defined and the immunization status of members of the population accurately recorded in the registry. We evaluated a city-wide immunization registry, focusing on its potential as a tool to study pertussis vaccine effectiveness, especially in adolescents.

**Methods:**

We conducted two evaluations – one in sites that were active registry participants and one in sites that had implemented an electronic medical record with plans for future direct data transfer to the registry – of the ability to match patients' medical records to registry records and the accuracy of immunization records in the registry. For each site, records from current pediatric patients were chosen randomly. Data regarding pertussis-related immunizations, clinic usage, and demographic and identifying information were recorded; for 11–17-year-old subjects, information on MMR, hepatitis B, and varicella immunizations was also collected. Records were then matched, when possible, to registry records. For records with a registry match, immunization data were compared.

**Results:**

Among 350 subjects from sites that were current registry users, 307 (87.7%) matched a registry record. Discrepancies in pertussis-related data were common for up-to-date status (22.6%), number of immunizations (34.7%), dates (10.2%), and formulation (34.4%). Among 442 subjects from sites that planned direct electronic transfer of immunization data to the registry, 393 (88.9%) would have matched a registry record; discrepancies occurred frequently in number of immunizations (11.9%), formulation (29.1%), manufacturer (94.4%), and lot number (95.1%.) Inability to match and immunization discrepancies were both more common in subjects who were older at their first visit to the provider site. For 11–17-year-old subjects, discrepancies were also common for MMR, hepatitis B, and varicella vaccination data.

**Conclusion:**

Provider records frequently could not be matched to registry records or had discrepancies in key immunization data. These issues were more common for older children and were present even with electronic data transfer. These results highlight general challenges that may face investigators wishing to use registry-based immunization data for vaccine effectiveness studies, especially in adolescents.

## Background

Population-based electronic immunization registries have the potential to reduce fragmentation of immunization records and to enhance immunization delivery [1,2]. Recently, much progress has been made in implementing registries at state, city, and regional levels [3,4]. The advent of electronic registries creates the possibility of using registry data to conduct vaccine effectiveness studies [5]. Registry-based studies could have several methodological advantages over traditional observational studies of vaccine effectiveness. First, the availability of individual-level immunization information for each person, or at least each child, in a population could allow large cohort studies to be conducted efficiently. This efficiency could reduce reliance on case control studies, which may be more prone to bias than cohort studies. Second, the ease of collecting immunization data from a registry also means that cohort studies could be conducted even in populations with low incidence of the vaccine-preventable disease under study. Studies conducted in outbreak settings may tend to underestimate vaccine effectiveness [6], so vaccine effectiveness studies conducted outside of outbreak situations are valuable in estimating the typical effectiveness of a vaccine. Third, the availability of complete and accurate information on the immunization status of residents of a registry's catchment area could also help avoid bias due to differential determination of immunization status among ill and well persons [7]. However, for a registry-based study to be valid, the base population must be clearly defined and the immunization status of the members of the population must be accurately recorded in the registry.

We conducted an evaluation of the Boston Immunization Information System, hereafter termed "the registry," focusing on its potential usefulness as a tool to study pertussis vaccine effectiveness, especially in adolescents. The registry has been operating since 1992. It was developed by the Boston Public Health Commission to support efforts to achieve and maintain high immunization coverage for infants and young children [8]. Boston has been remarkably successful in meeting this goal; its immunization coverage is among the highest of any large city in the nation [9]. Children are enrolled in the registry by their primary care providers, and provider immunization records are transferred regularly from participating primary care sites using customized software provided to each participating site. Sites have varied greatly in their technical capacity; early in the registry's history, some sites used manual data entry, which in some cases required double data entry, while others used electronic methods. At the time of this study, all sites used some form of electronic data entry and few required double data entry. Computing resources have improved over time, though, and, at the time of our evaluation, most sites were sending data directly to the registry from their own electronic records [8].

In many vaccine effectiveness studies, cases of disease are identified when patients with the vaccine-preventable disease are reported to a health department. In a registry-based vaccine effectiveness study, these cases would then be matched to registry vaccination records. Both the ability to match patients to registry records and the accuracy of those records would be important to the validity of the registry-based study. To simulate this process, we selected patients from pediatric primary care sites that participate in the registry and attempted to match these patients to registry records. Then, for children whose records could be matched, we evaluated whether discrepancies existed between the provider immunization record and the registry immunization record. We examined the kinds of discrepancies that occurred, to distinguish insignificant discrepancies from major ones that might limit the scope or accuracy of a vaccine effectiveness study. To enhance accuracy and efficiency, many registries and health care providers are moving toward direct transfer of immunization data to immunization registries from electronic medical records (EMRs). Therefore, we examined separately the expected completeness and accuracy of immunization data transmitted to the registry from providers using an EMR. Our assessment revealed several issues that could seriously limit the reliability of vaccine effectiveness studies using the registry. We believe that some of these problems may, to varying degrees, apply to other registries and have therefore summarized our findings and considered their general implications for investigators conducting registry-based vaccine effectiveness studies.

## Methods

The registry serves 23 health care sites that provide the great majority of the pediatric primary care for Boston, an urban setting with a 2000 population of about 116,000 children < 18 years old [10]. Only one practice site is private; all others are either in community health centers or in academic medical centers. This study consisted of two separate evaluations of the ability to match patients' medical records to registry records and the accuracy of immunization records in the registry. The first evaluation focused on pediatric primary care sites that were active registry participants, appraising the registry's current performance. This evaluation thus reflects the cumulative impact of all methods used for immunization data transfer to the registry over the history of the registry, through the time of the evaluation. The second evaluation focused on sites that had implemented a comprehensive EMR within 2–3 years before the evaluation. This second evaluation thus appraises the expected registry performance with direct transfer of immunization data from the EMR to the registry. The study was approved by institutional review boards serving all participating primary care sites, the Boston Public Health Commission, and the Boston University Schools of Public Health and Medicine. Only Boston Public Health Commission registry managers had access to individual registry records; registry data provided to Boston University investigators were either aggregated or de-identified.

### Collection of data from primary care providers

Pediatric primary care sites were identified by registry managers as 1) current registry users with good overall data integrity and completeness or 2) future direct-EMR-transfer users – former registry users that had temporarily stopped transferring data to the registry to allow implementation of the EMR with direct data transfer. All sites were invited to participate in the data quality review. Data were collected from August 2004 through February 2005 from sites that enrolled. For each primary care site, a random sample of pediatric records from current patients aged 0–10 and 11–17 years was chosen for review from the site's list of patients. For sites that reported serving < 5000 pediatric patients, 25 charts were chosen within each age group; for larger sites, 50 records were chosen within each age group. All components of each subject's medical record, including all volumes of paper records and all electronic records, were reviewed. For each subject, information on all immunizations containing diphtheria and tetanus toxoids was recorded; both those with and without pertussis antigens were included. We refer to these immunizations as "pertussis-related" hereafter. Data included the immunization date, formulation (DTwP, DTaP, DT, or Td; Tdap had not been introduced at the time of the study), manufacturer, and lot number. Additional data for each subject included 1) identifying information that might assist in matching records to the registry, including name, sex, date of birth, address, and mother's name; 2) dates of the patient's first visit to and first immunization at the primary care site; 3) for 11–17-year-olds, dates of MMR, hepatitis B, and varicella immunizations. When immunization data were recorded more than once in a subject's medical record, the data entered closest to the immunization date were considered to be the most accurate.

### Matching subjects to registry records

For primary care sites that were current registry users, subjects were matched to registry records by registry managers. A match was defined as occurring when a subject's provider record matched exactly one registry record with definite correspondence of at least name, sex, and date of birth. The spelling of names was not required to be identical. When more than one registry record existed for a given name, sex, and date of birth, other identifying data including immunization dates could be used to determine whether there was a match. For primary care sites that were future direct-EMR-transfer users, identifying data in the EMR would be expected to be identical to directly transferred registry data. Therefore, failure to match would be expected to occur only if a patient's paper medical record had not been updated into an EMR during the implementation of the EMR system. Therefore, for subjects from these sites, failure to match was considered to occur only when a subject did not have an EMR.

### Evaluation of immunization discrepancies between provider and registry records

For subjects from sites that were current registry users and who matched a registry record, registry managers evaluated whether discrepancies in immunization data existed between the provider record and the registry record. For each pertussis-related immunization, date discrepancies were categorized as 1–29 days difference, 30–364 days difference, or ≥ 1 year difference; formulation discrepancies were noted; and, for provider records with manufacturer and lot number information, any discrepancies in these fields were noted. Also, registry managers noted whether any discrepancies would have affected overall agreement between the provider record and the registry record as to whether the subject was currently up-to-date for pertussis-related immunization. For 11–17-year-old subjects, provider records of MMR, hepatitis B, and varicella immunization dates were compared to registry records using the same methods. To evaluate the sensitivity of the registry managers' evaluation of immunization discrepancies, artificial discrepancies were created in 10% of provider records and the detection of these discrepancies was tracked.

For subjects from primary care sites that were future direct-EMR-transfer users, immunization data in the EMR was considered to represent the data that would exist in the registry; the current EMR served as a proxy for the registry. Therefore, an immunization discrepancy was considered to have occurred when data in the EMR differed from data in the entire medical record, including all original volumes of the original paper record.

### Statistical analysis

We calculated frequencies of record matching and, among matched records, frequencies of discrepancies in immunization information for current user sites as a group, for future direct-EMR-transfer sites as a group, and for each site individually. We categorized both current age and age at first visit to the provider site into two categories – 0–10-years-old and 11–17-years-old. We then used the chi-square statistic to test differences in matching frequency. We used logistic regression to examine the independent contributions of current age, age at first visit to the provider site, and sex to 1) ability to match a registry record and 2) among matched records, discrepancy in whether the subject was up-to-date for pertussis-related immunization, constructing separate models for subjects from current user sites and from future direct-EMR-transfer sites. We analyzed the data with Excel 2003 and SAS version 9.1 (SAS Institute, Inc., Cary, NC).

## Results

### Population

Data were collected at 6 of the 8 pediatric primary care sites that were current registry users and at 7 of the 8 sites that were future direct-EMR-transfer users; the other 7 sites did not fit either category. Of 350 medical records selected at the 6 current user sites, 100% were available for review and abstraction of the demographic, clinical, and immunization-related data described above in the section on collection of data from primary care providers. Of 450 medical records selected at the 7 future direct-EMR-transfer sites, 442 (98.2%) were available.

### Matching

Among the 350 subjects from sites that were current registry users, 307 (87.7%) were definitively matched to a registry record, with a range among sites from 82.0% to 100%. Of these matched records, 300 (98.4%) were matched using only name, sex, date of birth, and, in some cases, address information; other identifying information was rarely needed. Among the 442 subjects from future direct-EMR-transfer sites, 393 (88.9%) would have matched a registry record once direct EMR transfer started, with the range among sites again 82.0% to 100%. An EMR did not exist for the remaining 11.1% of subjects from direct-EMR-transfer sites. These subjects had only a paper medical record and therefore would not have been represented in a registry populated by direct EMR transfer despite being considered current patients by the primary care site from which they were selected. Subjects who were 11–17 years old when they first visited their primary care site were less likely to match a registry record than subjects who were 0–10 years old at their first visit. This was true both at sites that were current registry users and future direct-EMR-transfer users (Table 1). In logistic regression models, age at first visit to the primary care site was the only statistically significant predictor of matching a registry record for both current user sites (adjusted odds ratio (aOR) for 0–10-year-old age group = 10.8, p = 0.0001) and future direct-EMR-transfer sites (aOR for 0–10-year-old age group = 4.0, p = 0.0005).

**Table 1:** Ability to match primary care provider records to registry records by current age and age at first visit to the primary care site.

	Current Registry Users			Future-Direct-EMR Users		
		
	Number with registry match/total	Percent with registry match	P-value*	Number with registry match/total	Percent with registry match	P-value*
Subject age group at time of review						
0–10 years	152/173	87.9	0.94	201/216	93.1	0.0067
11–17 years	155/177	87.6		192/226	85.0	
Age at first visit to primary care site						
0–10 years	274/301	91.0	< 0.0001	343/374	91.7	< 0.001
11–17 years	25/40	62.5		36/52	69.2	

* Chi-square test statistic

### Pertussis immunization discrepancies

From primary care sites that were current registry users, 288 matched records contained at least one immunization and so could be analyzed for accuracy of immunization data. In all, these records included 1410 pertussis-related immunizations (based on provider records, 522 DTwP, 464 DTaP, 135 DT or Td, and 289 with the formulation not recorded.) From future direct-EMR-transfer users, 377 matched records with 1848 pertussis-related immunizations were analyzed (based on provider records, 623 DTwP, 712 DTaP, 197 DT or Td, and 316 with the formulation not recorded). Table 2 presents data regarding discrepancies in all aspects of pertussis-related immunization. For subjects from current registry user sites, discrepancies were common in up-to-date status (22.6% of series had a discrepancy), number of pertussis-related immunizations received (34.7% of series had a discrepancy), dates (23.6% of series and 10.2% of immunizations had a discrepancy), and formulation (34.4% of immunizations had a discrepancy), with great variation between primary care sites. Manufacturer and lot number were rarely recorded in provider records and, when recorded, were all discrepant in the registry. For subjects from future direct-EMR-transfer sites, discrepancies were less common but still frequently present, especially for number of pertussis immunizations received (11.9% of series had a discrepancy), formulation (29.1% of immunizations had a discrepancy), manufacturer (94.4% of immunizations with manufacturer recorded in the provider record were missing manufacturer information in the registry), and lot number (95.1% of immunizations with lot number recorded in the provider record were missing lot number information in the registry.) Discrepancies were generally more common for 11–17-year-olds than for 0–10 year-olds (Table 3.) Logistic regression modeling showed that age at first visit to the primary care site was the only significant predictor of a discrepancy in up-to-date status for subjects from current user sites (adjusted odds ratio (aOR) for 0–10-year-old age group = 2.4, p = 0.03). Because very few discrepancies in up-to-date status occurred for direct-EMR-transfer sites, logistic models for this outcome could not be constructed for subjects from these sites.

**Table 2:** Discrepancies in pertussis-related immunization data between provider records and the immunization registry.

	Current registry user sites			Future direct-EMR-transfer sites		
		
	n	% with discrepancy	Range among provider sites	n	% with discrepancy	Range among provider sites
Discrepancies in pertussis-related immunization series						
Discrepancy in whether subject is up-to-date for pertussis-related immunizations	288	22.6	12.4–45.7	377	4.0	0–18.6
Discrepancy in number of pertussis-related immunizations received by subject	288	34.7	19.8–60.1	377	11.9	5.0–30.2
Any present in provider record but not in registry	288	27.8	11.9–60.9	377	0.3	0–1.2
Any present in registry but not in provider record	288	9.7	4.8–17.5	377	0.3	0–1.2
Discrepancy in date of any pertussis-related immunization in series	288	23.6	13.2–35.0	377	18.0	10.9–36.9
Discrepancy in formulation of any pertussis-related immunization in series	288	11.1	0–24.4	377	5.3	0–23.3
Discrepancies in pertussis-related immunizations						
Date discrepancy	1410	10.2	6.1–15.8	1848	8.0	5.5–17.0
Discrepancy of 1–29 days	1410	9.0	4.5–15.3	1848	6.6	3.0–16.5
Discrepancy of 1–11 months	1410	0.9	0–1.5	1848	0.8	0–2.0
Discrepancy of ≥ 1 year	1410	0.4	0–0.5	1848	0.5	0–1.5
Discrepancy in formulation		34.4	11.8–50.1		29.1	17.6–36.9
Discrepancy in whether pertussis-related immunization contained pertussis vaccine (vs diphtheria-tetanus only)	1410	0.6	0–2.0		0.4	0–1.2
Discrepancy in manufacturer, among those with manufacturer noted in provider record	468	100	NA	645	94.4	76.2–100
Discrepancy in lot number, among those with lot number noted in provider record)	539	100	NA	693	95.1	37.5–100

**Table 3:** Discrepancies in pertussis-related immunization data between provider records and the immunization registry by age group.

	% of subjects with discrepancy Current registry user sites		% of subjects with discrepancy Future direct-EMR-transfer sites	
		
	0–10 years old	11–17 years old	0–10 years old	11–17 years old
Discrepancies in pertussis-related immunization series				
Discrepancy in whether subject is up-to-date for pertussis-related immunizations	26.1	19.2	3.1	5
Discrepancy in number of pertussis-related immunizations received by subject	30.3	37.7	7.2	17
Any present in provider record but not in registry	23.2	32.2	0	0.6
Any present in registry but not in provider record	5.6	13.7	0	0.6
Discrepancy in date of any pertussis-related immunization in series	16.9	30.1	15.9	20.3
Discrepancy in formulation of any pertussis-related immunization in series	12.0	10.3	4.1	6.6
Discrepancies in pertussis-related immunizations				
Date discrepancy	4.9	14.3	5.5	10.2
Discrepancy of 1–29 days	3.8	13.1	4.4	8.5
Discrepancy of 1–11 months	1	0.8	0.8	0.8
Discrepancy of ≥ 1 year	0.2	0.5	0.2	0.8
Discrepancy in formulation	28.3	39.1	21.4	35.7
Discrepancy in whether pertussis-related immunization contained pertussis vaccine (vs diphtheria-tetanus only)	0	1.1	0	0.8
Discrepancy in manufacturer, among those with manufacturer noted in provider record	100	100	99.8	86.1
Discrepancy in lot number, among those with lot number noted in provider record)	100	100	99.8	89.0

### Discrepancies for adolescents in other immunizations

Table 4 presents data on discrepancies between provider records and registry records of MMR, hepatitis B, and varicella immunizations of 11–17-year-olds, based on 143 matched records with immunization data from this age group from current registry user sites and 180 from future direct-EMR-transfer sites. As for pertussis-related immunizations, discrepancies in up-to-date status, number of immunizations received, and dates were common overall and were more common in records from current registry user sites than from future direct-EMR-transfer sites. Varicella up-to-date status was especially likely to be discrepant.

**Table 4:** Discrepancies for 11–17-year-olds in MMR, hepatitis B, and varicella immunization data in provider records and the immunization registry.

	Current registry user sites N = 6 sites, 143 matched records		Future direct-EMR-transfer sites N = 7 sites, 180 matched records	
		
	% with discrepancy	Range among provider sites	% with discrepancy	Range among provider sites
MMR				
Discrepancy in whether subject is up-to-date for MMR immunization	19.6	9.5–43.5	14.4	5.3–36.4
Discrepancy in number of MMR immunizations received by subject	18.2	9.5–43.5	11.1	0–31.8
Discrepancy in date of any MMR immunization in series	22.4	5.3–33.3	16.1	7.5–38.1
Hepatitis B				
Discrepancy in whether subject is up-to-date for hepatitis B immunization	29.4	9.5–82.6	13.9	5.3–31.8
Discrepancy in number of hepatitis B immunizations received by subject	27.3	10.0–78.3	12.8	4.8–22.7
Discrepancy in date of any hepatitis B immunization in series	16.8	10.5–23.8	16.1	5.3–29.4
Varicella				
Discrepancy in whether subject is up-to-date for varicella immunization	69.2	23.8–100	63.9	57.5–82.4
Discrepancy in number of varicella immunizations received by subject*	30.4	20.0–80.0	7.6	0–20.0
Discrepancy in date of any varicella immunization in series*	3.6	0–10.0	2.9	0–20.0

*Excludes provider records with documentation of varicella disease.

### Sensitivity of registry review for discrepancies

Among 35 artificial discrepancies introduced into data abstracted from current registry user sites, 29 occurred in matched records. Registry managers detected 28 of those, for a sensitivity of 97%.

## Discussion

For several measures critical to vaccine effectiveness studies, this study showed that Boston's immunization registry would have significant shortcomings as a source of immunization data. Problems, discussed in more detail below, included inability to match subjects to registry records and incomplete and inaccurate immunization data. This registry is of one of the nation's oldest, so its data may reflect the cumulative impact of both population mobility and historical methods of data capture that are not manifest to the same degree in newer registries. This interpretation is supported by our finding that data for adolescents were generally less reliable than for younger children. Also, reflecting the delivery of pediatric care in Boston, almost all sites participating in this registry are community health or academic medical centers. Nationwide, however, most immunizations are delivered in the private practice setting, with processes and documentation that may differ from those used in the Boston sites. We doubt that the problems revealed in our study are unique to this registry, however. Previous studies of registries focusing on vaccination delivery and coverage, rather than on vaccine effectiveness, have shown varying but suboptimal completeness and accuracy [11-15]. Although the problems we found may (or may not) be particularly severe in this registry, our findings highlight general challenges that may face investigators wishing to use registry-based immunization data for vaccine effectiveness studies.

Matching persons identified with cases of the vaccine-preventable disease under study to their registry immunization records is likely to be a first step in many registry-based vaccine effectiveness studies. In this registry, matching was incomplete in all age groups and was particularly problematic in children who were in the 11–17-year-old age group at their first visit to their primary care provider. We do not know whether these children moved to Boston from another location or transferred from one provider to another within the city, but the finding provides a cautionary note for the use of registry data in studies of adolescents. It might be tempting to include persons with disease in a vaccine effectiveness study regardless of whether they match a registry record, since their immunization records are likely to be available from other sources. However, doing so could bias the estimate of vaccine effectiveness, if the vaccination status of persons who match a registry record is systematically different from that of persons who do not match. For example, persons with a matching registry record might be, on average, more completely vaccinated than persons without a matching registry record. If so, then a study including diseased persons who do not match a registry record would upwardly bias vaccine effectiveness estimates, because these cases would be less likely to be completely vaccinated than the population represented by the registry independent of any protective effect of the vaccine.

We suspect that two main factors, both with implications for vaccine effectiveness studies, account for the lower registry matching rates of older children. First, the registry was launched in 1992, meaning that the oldest patients enrolled during infancy would have been 13 years old during our evaluation; patients older than 13 were necessarily enrolled at older ages with retrospective entry of their historical immunization data. Vaccine effectiveness studies in adolescents are of current interest, as several new vaccines have recently been recommended for this age group [16-18]. If subjects in a registry-based study are older than the registry, investigators will need to consider how historical data were handled. Second, the US population has high rates of moving between states or regions [19]; even registries that automatically enroll children at birth could be prone to incomplete matching of children who move into the registry catchment area later in life. Direct transfer of data from EMRs into the registry might be expected to solve the problem of incomplete matching, at least for children served by providers using EMRs. Interestingly, though, using EMR data as a proxy for registry data and comparing these data to the entire medical record, we found that similar rates of failure to match – slightly lower in 0–10-year-olds and slightly higher in 11–17-year-olds – would be expected for children from sites using an EMR. This phenomenon occurred because most provider sites did not create an EMR for all of their existing patients during implementation of the EMR system; a registry record would only be generated through direct data transfer for patients with an EMR.

Patterns of immunization data discrepancy for children who did match a registry record also have important implications for vaccine effectiveness studies. Most discrepancies in the number of pertussis-related immunization were due to immunizations that were recorded in the provider record but were absent from the registry (Table 2), implying that the registry record, not the provider record, was incomplete. We also found that the data on vaccine formulation, manufacturer, and lot number were often either absent from the provider record or, when present, discrepant in the registry. These types of discrepancies have minimal consequences for achieving the registry's primary goal, supporting efforts to achieve and maintain high immunization coverage among young children, aside from time lost in tracking children who appear incorrectly to have immunization delay. For vaccine effectiveness studies, however, these discrepancies could have important implications. The importance of using comparable methods to determine vaccination status of diseased and non-diseased subjects has been emphasized previously [7]; for registry-based studies, this principle implies that, if the registry is the sole source of vaccination information for non-cases or the population as a whole, then it should be the sole source for cases also. Even if immunization data for all study subjects is obtained from a registry, however, to the extent that some immunizations are missing from the registry (and that the pattern of missing immunizations is not related to disease status), vaccine effectiveness estimates will be biased toward the null. Immunization data that are captured and transmitted to a registry from EMRs or by other electronic means should be more accurate than data transmitted by other methods [20,21], but our data show that, beyond the errors that can occur during actual use of electronic medical records, much information can be lost from paper records during the implementation of the electronic system. Investigators should therefore consider the impact of all the methods of data entry used over a registry's history for studies in which data for any immunizations under study would have been entered using past methods.

Before relying on registry data for a registry-based vaccine effectiveness study, therefore, we suggest that it would be prudent to conduct an evaluation of the registry's population coverage and data quality. We recognize, however, that conducting such an evaluation may in itself be challenging. Registries are intentionally and appropriately designed with a high level of confidentiality for participating patients and provider sites and so may be prohibited from releasing to investigators the individual-level data needed to conduct such an evaluation [1,2,22,23]. In our case, compliance with registry confidentiality policies meant that we needed to develop a somewhat cumbersome method for reviewing data abstracted from provider records that was labor-intensive for registry managers. However, a registry-based vaccine effectiveness study conducted without this evaluation would almost certainly have produced misleading results.

## Conclusion

In summary, our evaluation revealed limitations with registry data – frequent inability to match provider records to registry records as well as frequent discrepancies in key immunization data – that have implications both for defining study populations and for conducting data collection for vaccine effectiveness studies. These issues were more common for older children and were present even with electronic data transfer. Registry characteristics required to support immunization delivery do not necessarily translate into complete and accurate vaccination information for the population as a whole. These results highlight general challenges that may face investigators wishing to use registry-based immunization data for vaccine effectiveness studies, especially in adolescents. The frequency of incomplete and inaccurate information will certainly differ between registries, but we believe the qualitative lessons learned from this evaluation are likely to be applicable to other registries.

## Competing interests

The authors declare that they have no competing interests.

## Authors' contributions

BEM conceived of and designed the study, conducted the literature review, supervised and participated in data collection, directed the data analysis, and drafted the manuscript. KMS participated in data collection, designed and managed the study database, performed the statistical analysis, and contributed to writing the manuscript. NND participated in study design and data collection and revised the manuscript. AML participated in study design and data collection, contributed to data analysis and interpretation, and revised the manuscript. All authors read and approved the final manuscript.

## Pre-publication history

The pre-publication history for this paper can be accessed here:


